# The Frequency and Fatality of Hæmorrhage in Gastric Ulcer

**Published:** 1901-04-20

**Authors:** 


### THE FREQUENCY AND FATALITY OF HÆMORRHAGE IN GASTRIC ULCER.

In reference to the dispute as to the importance of hæmorrhage as a complication of gastric ulcer Mr. Mayo Robson [1] formulates his contention as follows. He argues (1) that 5 per cent. of the population suffer, or have suffered, from gastric ulcer; (2) that 25 per cent. of those suffering from gastric ulcer die directly or indirectly from the disease; (3) that 50 per cent. of cases of gastric ulcer bleed; and (4) that 7 per cent. of cases of hæmatemesis from ulcer, or 3½ per cent. of all persons suffering from ulcer, die as the result of the bleeding. Working this out for a population of 500,000, and spreading the death-rate from this cause over an average life of 50 years, Mr. Mayo Robson concludes that 17·5 patients die per annum in such a population from hæmorrhage due directly or indirectly to ulcer of the stomach. If this is so, and if the experience of others should support that which he has detailed as to the efficacy of surgical intervention, it is clear that the whole question of treatment in such cases will have to be reviewed.

[1] Lancet, April 13.

